# The Effect of Human Error on the Temperature Monitoring and Control of Freeze Drying Processes by Means of Thermocouples

**DOI:** 10.3389/fchem.2018.00419

**Published:** 2018-10-01

**Authors:** Micaela Demichela, Antonello A. Barresi, Gabriele Baldissone

**Affiliations:** Department of Applied Science and Technology, Politecnico di Torino, Turin, Italy

**Keywords:** freeze-drying, temperature monitoring, operational error, risk assessment, product quality, operational safety margins

## Abstract

Monitoring product temperature is mandatory in a freeze-drying process, in particular in the process development stage, as final product quality may be jeopardized when its temperature trespasses a threshold value, that is a characteristic of each product being freeze-dried. To this purpose thermocouples are usually inserted in some of the vials of the batch to track product dynamics. The position of the thermocouple inside the vials strongly affects the reading of the temperature evolution during the freeze-drying process and, thus, it is necessary to place them in the right position, in such a way that correct information about product temperature is obtained. In this work, at first, the probability of the operational error resulting into a wrong positioning of the thermocouple inside the vial has been estimated experimentally. Then, the effect of this error has been assessed in terms of risk of exceeding the limit temperature in the primary drying step. Both 4R and 10R vials have been considered, and the investigation evidenced that the probability of incorrect thermocouples placement can reach 30% for 10R vials, and about 32% for 4R vials. These probability values increase, respectively, to 47 and 39% when the trays containing the vials are shifted to their final position. Then, through IR thermal imaging it has been possible to evaluate the temperature gradients in a vial, pointing out that the temperature difference between the product at the center of the vial, where the thermocouple is supposed to be, and that of the wall, that is quite often measured by the thermocouples, can be about 1°C. Therefore, associated to each thermocouple reading there is a probability distribution of product temperature. These figures can be used to assess the risk of exceeding the limit temperature in a freeze-drying process and, thus, to quantify suitable safety margins when evaluating thermocouple readings to take into account the operational errors, given a risk tolerability criteria.

## Introduction

Vacuum freeze-drying is a process widely used in the pharmaceutical industry to remove a liquid from a product, usually a solvent from a solution containing the active pharmaceutical ingredient and the excipients. The product is firstly frozen and, then, the solvent used (generally water, although water-organic solvents are also employed for certain products) is eliminated by sublimation. As it is carried out at low temperature, with direct sublimation of the frozen solvent, it allows to avoid the thermal stresses to the active principle which may be typical of other drying techniques. Besides, it can be carried out in sterile conditions, which is essential for parenteral products, it allows fast reconstitution of the final product, and, generally, it assures a long shelf life, thus eliminating the necessity of maintaining the cold chain.

Tight temperature control is essential already in the freezing step, because the structure of the porous matrix is determined by the freezing protocol (and, in particular, by cooling rate, freezing rate, and temperature and duration of the holding time). In particular, in order to obtain a good uniformity of the properties of the batch it is essential that ice nucleation and freezing is completed in a small temperature interval. Even more important is the control of the temperature during the drying time, both during primary drying, when it is necessary to avoid melting or collapse of the product, and during secondary drying, when scorch of the surface can occur, especially in case of radiant heating. In fact, even if freeze-drying is considered a “gentle” process, the collapse temperature of several typical pharmaceutical excipients may be quite low; in addition, as the residual moisture content typically affects it negatively, the product limit temperature may be very low during primary drying because, even after the ice sublimation, significant amounts of bound water can be present in the product. On the other hand, heat must be supplied because the process is endothermic, and the shelf temperature must be optimized in order to reduce at a minimum the process duration.

As manipulated variable, generally, the shelf temperature is selected in industrial practice; a control system is used to keep the temperature of the heat transfer fluid (generally a silicon oil), passing internally through the shelves, at the set point value. It must be said that heat transfer control obtained through the manipulation of shelf temperature is slow, due to thermal inertia of the system, and shelf heating and cooling may induce a large lag in product temperature response. Alternatively, the chamber pressure can be manipulated: this is a very responsive system, because the heat flux from shelf to product strongly depends on chamber pressure, but is also very risky, because the product temperature practically follows the pressure variations, and changes of few pascals can jeopardize the product quality.

In the perspective of obtaining “Quality-by-Design” it is essential to adopt a closed loop control policy (Barresi and Fissore, 2011). This subject has been widely investigated and several control systems were proposed in the past using the product temperature measurement as monitoring tool (Barresi et al., 2018), but up to date it has found a limited application in the pharmaceutical industry, where more often a regulation approach (or open-loop control) is adopted: that is, the shelf (or transfer fluid) temperature is monitored and maintained at the set-point value. Very often, anyway, the product temperature is also monitored, and this monitoring can allow evidencing if some failures in the pressure control (with consequent pressure increases) occurred. On the contrary, product temperature monitoring and control is widely used in laboratory scale and in the process development stage.

In any case the correct product measurement is crucial. In case of closed loop control, in fact, it directly affects the control action. But even more serious may be the effect of errors in the cycle development stage, as a shelf temperature sequence will be developed as a consequence of the product temperature readings (and model parameters identification, heat transfer coefficient and cake resistance, as discussed below), that will be then applied to production cycles with a open loop control.

Detailed reviews of the currently available measuring devices for freeze-drying monitoring can be found in literature (Patel and Pikal, 2009; Patel et al., 2010; Nail et al., 2017; Fissore et al., in press). Different methods have been proposed also for measuring the product temperature; some of them, based on the pressure rise test, allow estimating the average value of the batch, but thermocouples, or resistance thermal detectors (RTD), inserted in the vial, in contact with the product, are the most widely used. Thermocouples are generally used in lab-scale freeze-dryers (Fissore et al., 2017): if thin wires are chosen, the sensing tip can be very small, and this allows a punctual temperature measurement and an easier and more accurate positioning in the batch, but, of course, the measure becomes very sensitive to the correct location, and even a very small displacement can affect the result; human and operational errors can thus become relevant.

RTDs are most frequently used in production freeze-dryers as they are more robust and can be sterilized (Willemer, 1991; Oetjen and Haseley, 2004); this larger sensing device measures a larger portion of the product, giving an average value, less accurate, but more robust. In general, a good compromise must be found, considering also the mechanical robustness of the device and of the wires (Nail et al., 2017). The Temperature Remote Interrogation System (TEMPRIS) sensors, passive transponders which receive energy from an electromagnetic field, thus eliminating the necessity of wire connections, are another type of device recently proposed (Schneid and Gieseler, 2008). Unfortunately, the size of this device is so large that can cause significant modifications of the total volume, or cause uncontrolled freezing even if not immersed, making the temperature measurement not very reliable.

It must be considered that at a certain point during the primary drying stage the temperature monitored by the thermocouples starts increasing rapidly up to the heating shelf temperature. This may be due to the loss of contact between the sensor tip and the product, or to the fact that the interface of sublimation passes the sensor tip (Bosca et al., 2013a). Therefore, the temperature measurement may be used for product monitoring and process control only in the first part of the primary drying stage. In any case, it has to be considered that, after the initial transient, product temperature reaches a sort of steady-state (as all the heat received is used for ice sublimation) and, thus, the operating conditions have to be optimized only in the first half of the primary drying stage (Bosca et al., 2013b).

It must be noted that the product temperature measurement is widely employed not only to monitor the process, but also for the identification of model parameters, very useful for process development and cycle optimization. The heat transfer coefficient *K*_*v*_ may be calculated using the following equation, depending on the temperature measured at the bottom of the vial (*T*_*B*_), obtained from the heat balance for the frozen product:

(1) $$\begin{array}{r} K_{v}=\frac{m\Delta H_{s}}{A_{v}\int_{0}^{t_{drying}}\left(T_{shelf}-T_{B}\right)dt} \end{array}$$

where *m* is the mass of ice in the vial, *A*_*v*_ is the cross-section area of the vial, *t*_*drying*_ is the time required to complete the ice sublimation and Δ*H*_*s*_ is the heat of sublimation. If the temperature measurement is not available up to the end of the primary drying step, it may be assumed that the slope of the temperature profile does not change if the temperature of the heating source and the pressure in the chamber are not modified. Once *K*_*v*_ is known, also the cake resistance to water vapor flow, *R*_*p*_, can be obtained (Fissore et al., 2015). A procedure for rapid determination of dry layer resistance to various pharmaceutical formulations during primary drying using product temperature profiles has been also proposed by Kuu et al. (2006).

As an alternative, the thermocouples can become a very powerful Process Analytical Technology (PAT) tool, using a soft-sensor. This is an algorithm which estimates in-line the interface temperature, *T*_*i*_, and *K*_*v*_ using the measured value of temperature at the bottom of the product, *T*_*B*_, and calculates *R*_*p*_ and *L*_*dried*_, that is the thickness of dried cake. Thus, it is possible to monitor inferentially also the drying process and the position of the interface (Bosca et al., 2015), or estimating the cake resistance (Bosca et al., 2013a). Obviously, using multiple sensors, placed in representative positions of the batch, it is possible to take into account its non-uniformity (Bosca et al., 2013b).

As the position of the sensing device affects the actual reading, the correct and precise positioning of the sensor is of great importance for the accuracy of the measure, as there are temperature gradients in the product and the tip of the device must be in the frozen product. In the case of vials, the best position is in the center, very close to the bottom, and positioning devices may be helpful and are recommended; in case of bulk products, on the other hand, the positioning of the probe may be problematic (Nail et al., 2017). It is evident that the role of human factors becomes relevant as well as the analysis of technological failures, since the positioning of the probe, and its maintenance, is still a manual operation.

The present work is aimed at estimating experimentally, performing visual checks, the probability of the operational error resulting into a wrong positioning of the thermocouple inside the vial. The maximum error in temperature reading has been estimated experimentally, evaluating the temperature gradients in a vial through IR thermal imaging. Analysis focused, in particular, on the temperature difference between the product at the center of the vial (where the thermocouple is supposed to be) and that at the wall (that can be measured by the thermocouples in case of maximum deviation from correct positioning); the probability distribution of product temperature associated to each thermocouple reading has been also assessed. This allows the evaluation of the risk of exceeding the limit temperature in a freeze-drying process.

## Operational error estimation

Wherever an operation involving human intervention must be performed, the occurrence of an operational error must be taken into account. In the present case, the human error may be related to the thermocouple positioning, that can affect the results of a measurement, on which the monitoring and control of a process has to rely on. According to Jacob et al. (2013) measurement error may occur because the measurement system is not accurate enough or precise enough, or because of an operational error of the operator involved in the measurement, and an approach to estimate and reduce them has been proposed accordingly.

More in general, several models have been developed during the years to assess human errors and to identify their causing and influencing factors. A review of these methodologies can be found in Boring et al. (2010), while in Petruni et al. (in press) a methodological support to choose among them is proposed.

In the present case, more than the analysis of the causal factors, the aim was to assess the probability of occurrence of an improper positioning of the thermocouple in the vial, and to estimate its effect on the monitoring of the freeze-drying process. Thus, a simpler methodology was adopted, analyzing from a statistical point of view the errors committed during a set of experiments. In particular, as discussed in Kotek and Mukhametzianova (2012), the operation of positioning the thermocouple in the vial has been repeated by different operators in an experimental setup described below.

From the observations a point error probability has been obtained, according to the following equation:

(2) $$\begin{array}{r} Pr_{error}=\frac{n_{erroneous\;ops}}{N_{tot\;ops}} \end{array}$$

where *Pr*_error_ is the point probability of an incorrect positioning of the thermocouple, *n*_erroneous ops_ is the observed number of incorrect positions and *N*_tot ops_ is the total number of positioning operations.

The observed data have been then interpreted to identify a suitable distribution, allowing to predict errors for future operations and to estimate the risk of exceeding the limit temperature in the primary drying phase, thus identifying the safety margins in the temperature monitoring and control. The results are discussed in a dedicated section.

### The experimental set-up

**Figure 1:**
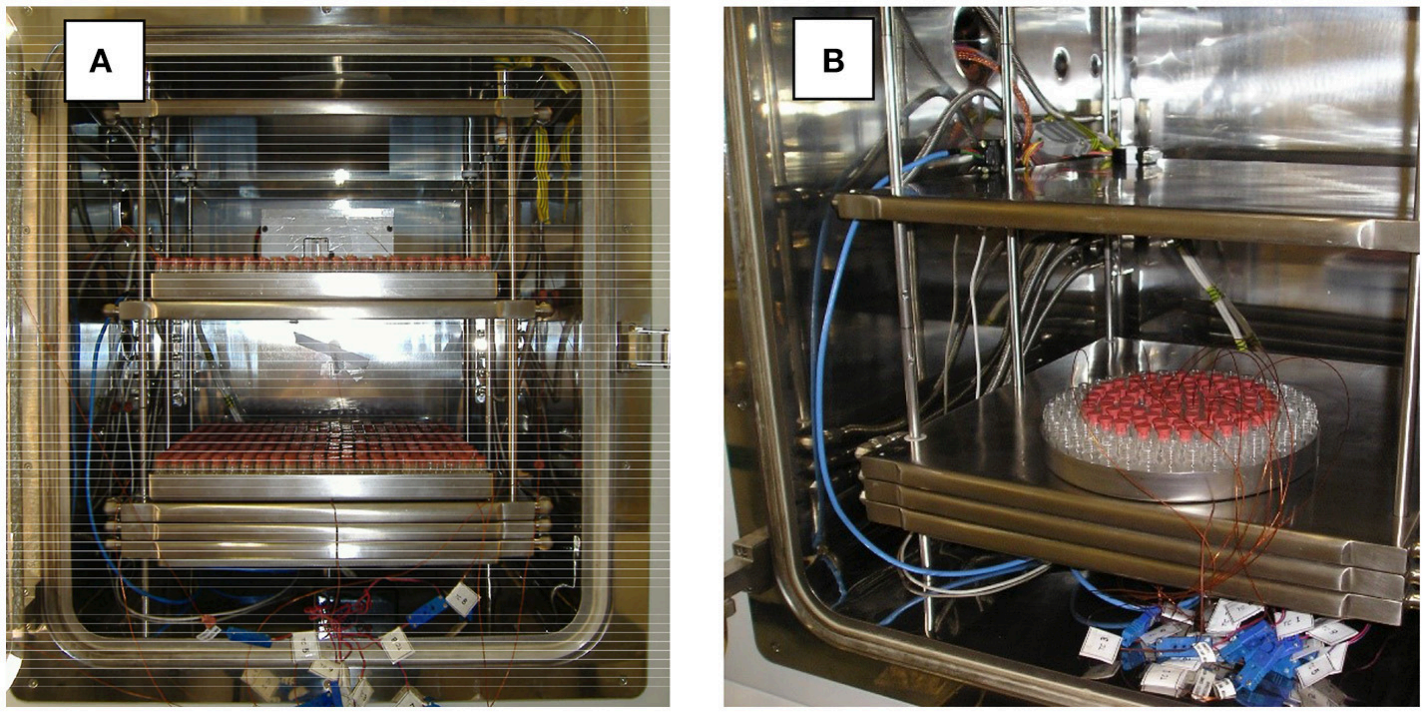
Typical vial arrangements in a freeze-dryer, during the cycle development stage, with thermocouples inside some vials for process monitoring. The cases of large rectangular trays **(A)** and a smaller circular tray, with empty vials in the external rows for shielding purposes **(B)**, are shown.

When the real operations are performed, a batch is made of one or more trays filled with ordered vials. Usually 5–7 thermocouples are placed in the vials to keep under control the temperature evolution throughout the freeze-drying operation. Typical setups are shown in Figure 1.

To simulate the procedure of the thermocouples positioning, 10 vials have been used, with rubber plug, in which the thermocouples must be inserted. T-type miniaturized thermocouples having a resolution of about 0.1°C have been employed; the thermocouples have a very thin exposed tip, isolated wires, and are relatively flexible, with a 0.5 mm overall diameter.

**Figure 2:**
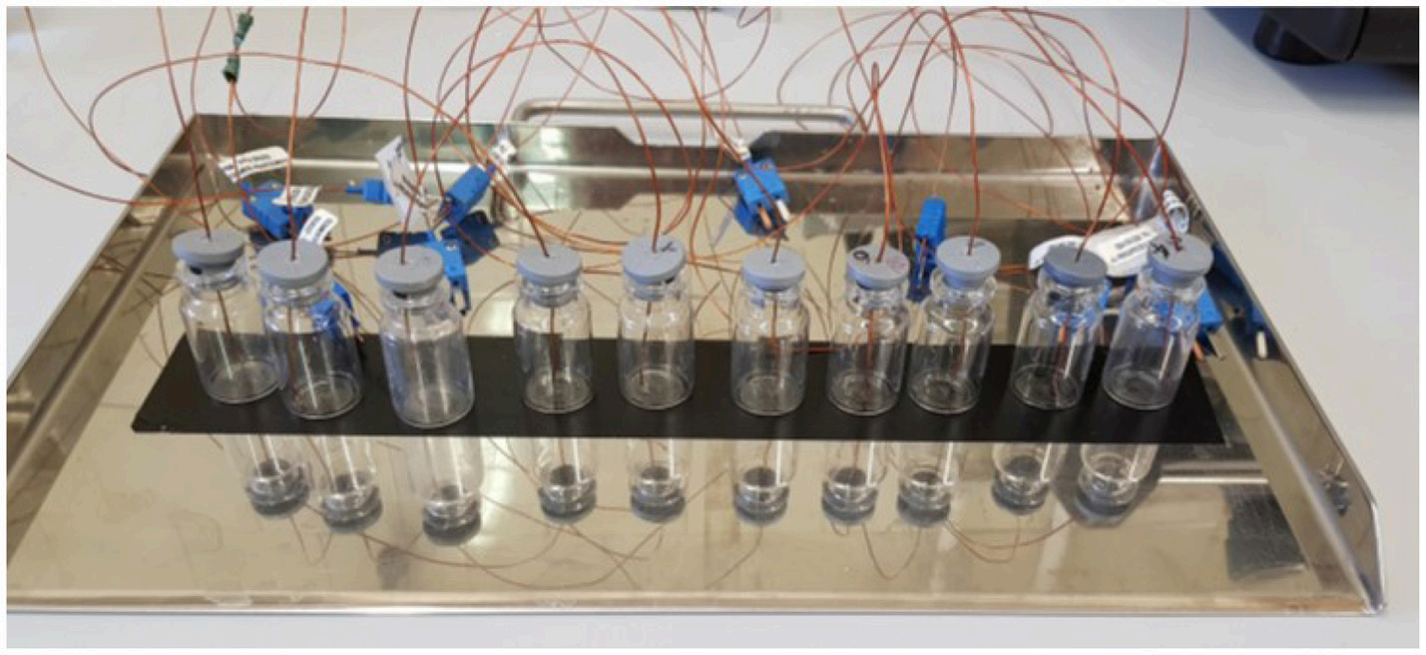
The experimental setup used for testing the thermocouple insertion procedure; here a realization with 10R vials (24 mm diameter) is shown.

Since in the tray the vials stability is guaranteed by the presence of adjacent vials, in the experimental setup an adhesive stripe has been used to avoid that the thermocouple weight could unbalance the vial itself. Figure 2 shows the experimental setup used in this study.

Vials of two different sizes have been tested, namely 4R, with a diameter of 16 mm, and 10R, with a diameter of 24 mm. The operators had to insert the thermocouple in the plug, place the plug on the top of the vial, leaving enough space for vapor release during the drying step, and, then, adjust the position of the thermocouple and of the plug in order to place the sensing element in the correct position: at the bottom of the vial, in its center. Then, a check of the thermocouple position was made through visual inspection. Afterwards, the tray has been moved to another position, in order to simulate the positioning of the tray inside the equipment and to verify if some change of position could occur. The tray movement was carried out reprodicibly by the same trained reasearchers.

Twelve operators carried on the test on 4R vials and eleven on 10R vials: in each test the operator had to position ten thermocouples, one for each of the 10 vials available. Only two of the operators were already trained for the operation, highlighted in bold in Tables 1, 2 where the results of the tests are summarized. The untrained operators have been informed about the operation to be carried on through a 5 min presentation, describing the operation to be carried on and the possible consequences of an uncorrect positioning, followed by the visualization of the operation carried on by trained operators.

**Table 1:** Thermocouples positioning in 4R vials–after tray shift.

**Test**	**Vial**									
	**1**	**2**	**3**	**4**	**5**	**6**	**7**	**8**	**9**	**10**
1	C	C	C	W	W	U	W	C	W	W
**2**	**C**	**C**	**C**	**C**	**W**	**W**	**C**	**W**	**C**	**C**
3	C	C	U	C	C	C	C	C	C	C
4	W	C	C	W	W	C	C	C	C	C
5	C	C	W	C	C	C	C	C	C	W
6	C	C	W	C	U	C	C	C	W	W
7	C	W	W	C	C	W	W	W	C	C
8	W	C	C	C	W	C	C	C	C	C
9	W	C	W	C	W	W	W	W	C	C
10	W	W	W	W	W	W	C	C	C	C
11	C	W	W	W	C	W	W	C	C	C
**12**	**W**	**C**	**W**	**C**	**W**	**W**	**C**	**C**	**C**	**C**

*C, correct; W, wall; U, up*.

*Gray cells indicate cases were position changed after the shift*.

**Table 2:** Thermocouples positioning in 10R vials–after tray shift.

**Test**	**Vial**									
	**1**	**2**	**3**	**4**	**5**	**6**	**7**	**8**	**9**	**10**
1	W	W	C	W	C	W	W	C	C	C
2	C	C	C	C	W	W	C	C	C	W
3	C	C	W	C	C	C	C	C	W	W
4	C	W	C	W	W	W	W	W	W	C
5	W	W	W	W	W	W	C	C	W	W
6	W	C	C	W	W	C	C	C	C	C
7	C	C	W	W	C	C	W	C	C	C
8	C	C	W	W	C	C	C	C	C	W
9	W	C	C	W	C	C	W	W	W	C
10	C	W	W	C	C	C	W	C	C	C
**11**	**C**	**W**	**W**	**C**	**C**	**C**	**W**	**C**	**C**	**C**

*C, correct; W, wall; U, up*.

*Gray cells indicate cases were position changed after the shift*.

The time required to conclude the test has not been imposed. A mean time of about 7 min has been observed in preparing 10 vials, with no relevant differences between the time to prepare the 4R vials (6′48″) and the 10R ones (7′10″), and between expert and non-expert operators.

### Experimental evaluation of temperature gradients in vials

Freeze-drying tests were carried out using a LyoBeta 25™ (Telstar, Spain) freeze-dryer (drying chamber: 0.2 m^3^, total shelf area: 0.5 m^2^).

Temperature profiles inside the vials were evaluated by infra-red imaging, by a specially designed system (IMC Service S.r.l., Italy), including a thermal camera (FILR Systems model A35). Emissivity of the glass vials (0.9, measured from the producer according to ISO 18434-136 guideline), distance from the subject, reflected apparent temperature, room temperature and humidity, were taken into account; inserted thermocouples were employed for the validation and calibration procedure. Details of the apparatus and set up, calibration and validation procedure can be found in the work by Lietta et al. (in press).

## Results and discussion

**Figure 3:**
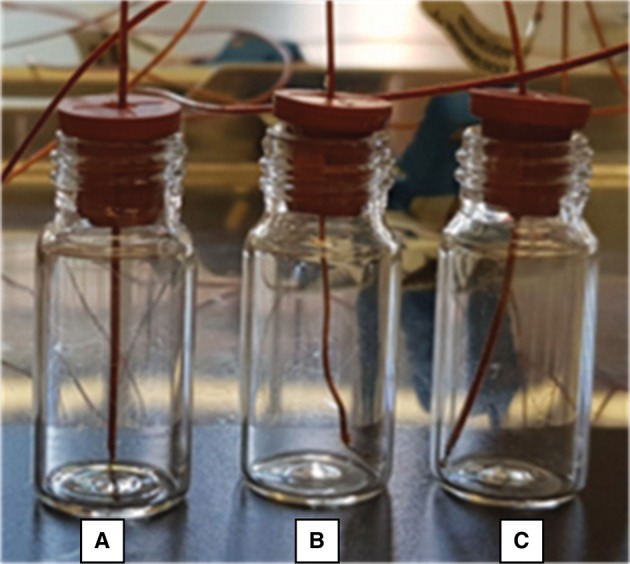
An example of the possible cases observed about thermocouple positioning: **(A)** correct placement, close to the bottom center; **(B)** lifted thermocouple with respect to vial bottom; **(C)** incorrect positioning, with the sensing tip in contact with the lateral wall.

Table 1 shows the results of the observations of the thermocouples positioning in 4R vials after the shift of the tray. 120 vials have been equipped with the respective thermocouple: before the tray shift 82 have been placed in the correct position, after the shift only 73 remained in the correct position. Three main cases have been observed: the correct positioning, corresponding to the sensing element located in the center of the vial bottom, in contact with the vial (Figure 3A), recorded in the Tables with the letter “C”; an incorrect positioning, with the sensing element lifted from the vial bottom (Figure 3B), recorded with a letter “U” (for “up”); and an incorrect positioning, with the sensing element in contact with the vial, but on the lateral wall (Figure 3C), recorded with the letter “W” (for “wall”). The gray cells in Table 1 represent those thermocouples that, after the tray shift, changed their position. The quite low numbers of grayed cells (< 10%) can be related to the fact that the rubber plugs of smaller vials had, by structure, a greater rigidity to the thermocouple cable. It has also to be noticed that the thermocouples in the “U” position (Figure 3B) are only two and, thus, it was decided to include these cases within the other incorrect positions.

Table 2 shows the results of the observation of the thermocouple positioning in 10R vials, after the shift of the tray to another position. 110 vials have been equipped with the respective thermocouple: before the tray shift 77 have been placed in the correct position, after the shift only 64 remained in their original correct position. Again, the gray cells in Table 2 represent those thermocouples that following the tray shift changed their position. The higher numbers of grayed cells (a little higher than 10%) can be related to the less tightening structure of the rubber plugs of larger vials. No thermocouples in the U position have been observed in this type of vials.

From the observations above reported it is possible to assess the point probability of having the thermocouple in the wrong position, according to Equation (2). Table 3 shows the point probabilities of incorrect position, before and after the tray shift. From the data it is possible to argue that the probability of error, more related to a so called “commission error” in positioning the thermocouple, is similar for the two types of vials, although the 10R vials are more sensitive to the shift of the trays. This last type of error is clearly more related to an “omission error,” due to the lack of control of the operator after the tray shift, that could correct the thermocouple position.

**Table 3:** Observed point probability of incorrect positioning of the thermocouple in the vials.

**Vial type**	**4R**			**10R**		
	**Vial number**	**Incorrect position**	**Probability**	**Vial number**	**Incorrect position**	**Probability**
Before tray shift	120	38	0.317	110	33	0.300
After tray shift	120	47	0.392	110	56	0.467

The lack of control after the tray shift may be well-representative of the real process, where it is difficult to verify the position of the thermocouples in the freeze-dryer during loading. The conditions experienced by the operators during the test are similar to the conditions in a real process, even if probably a little less stressed and simplified, as empty vials have been used, with no time constraints. In real cases, depending on the product characteristics, the liquid must be kept below a given temperature, and there may be time constraints, consequence of the limited stability of the product after formulation and filling, which can increase the stress on the operators, consequently increasing the chances for errors.

The collected data allow to identify a distribution of error probability, in terms of number of incorrect positioning over 10 insertions.

**Figure 4:**
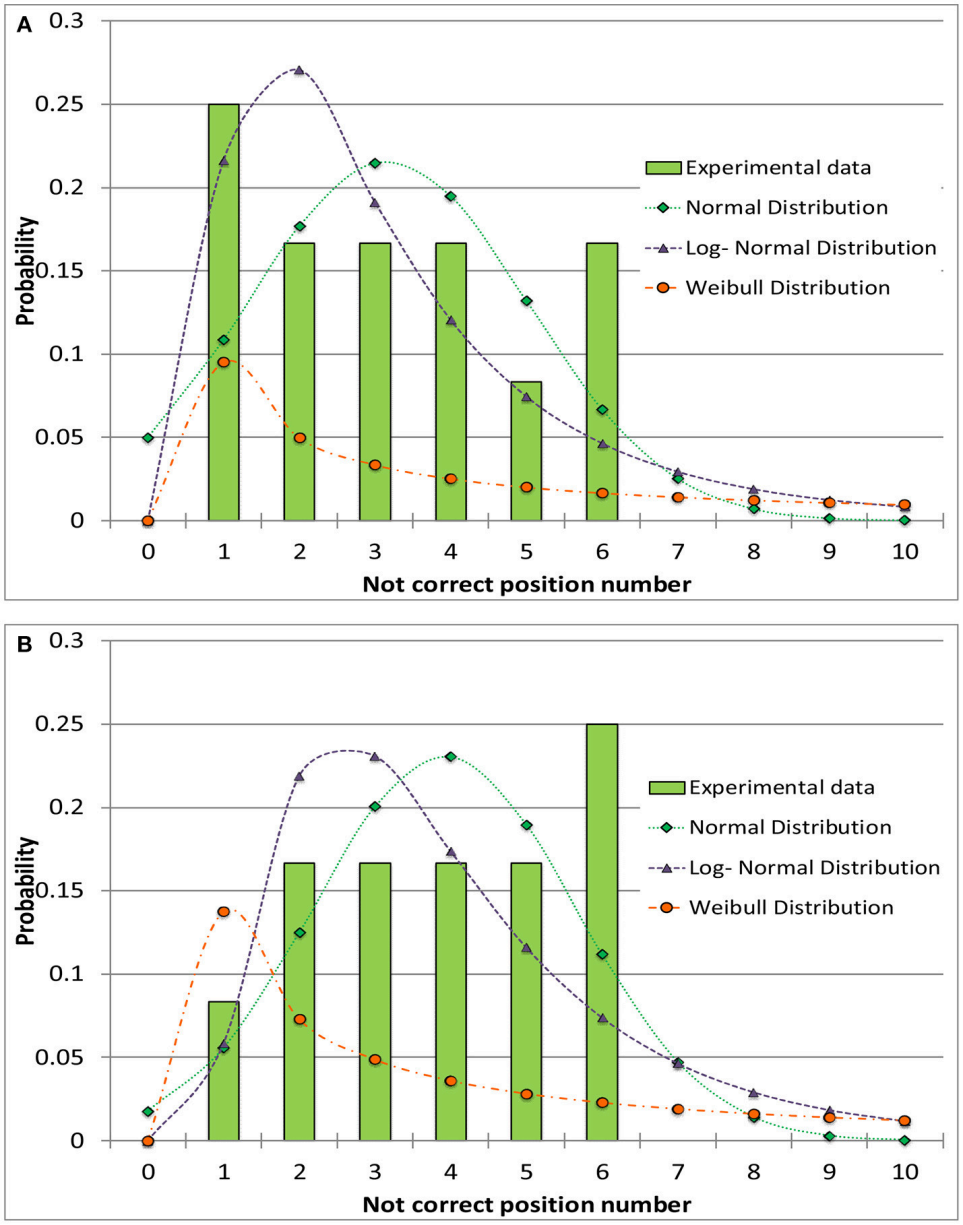
Comparison between different distribution functions and the experimental data for the incorrect positioning of thermocouples in the 4R (16 mm diameter) vials. **(A)** Before the shift of the tray. **(B)** After the shift of the tray.

The experimental data were approximated through probability distribution functions: normal, log-normal and Weibull distribution have been tested. The Weibull distribution was found to be the poorest one in fitting experimental data. The normal and log-normal distributions showed similar fitting performances of the experimental data (adopting the minimum mean square error criterion). An example of a data set modeling is shown in Figure 4 for the 4R vials; similar results have been obtained for the 10R vials. The calculated cumulative mean square errors reported in Table 4 for the normal and the log-normal distribution are similar, even if small differences are observed for the cases before and after the tray shift. In detail, the normal distribution better represents the case with higher number of errors and better describes the increase of the error probability after the tray shift (Figura 4B). For this reason the normal distribution was chosen in this case. It is represented by Equation (3), where *f(x)* is the probability density at the point *x*, where the probability is evaluated, μ is the mean value of the distribution and σ is its standard deviation:

(3) $$\begin{array}{r} f\left(x\right)=\frac{1}{\sqrt{2\pi \sigma^{2}}}e^{-\frac{{\left(x-\mu \right)}^{2}}{2\sigma^{2}}} \end{array}$$

**Table 4:** Fitting quality data (evaluated by the mean square error) for the 4R type vials.

**Distribution type**	**Normal**	**Log-normal**	**Weibull**
Before tray shift	0.039	0.031	0.102
After tray shift	0.030	0.045	0.114

**Figure 5:**
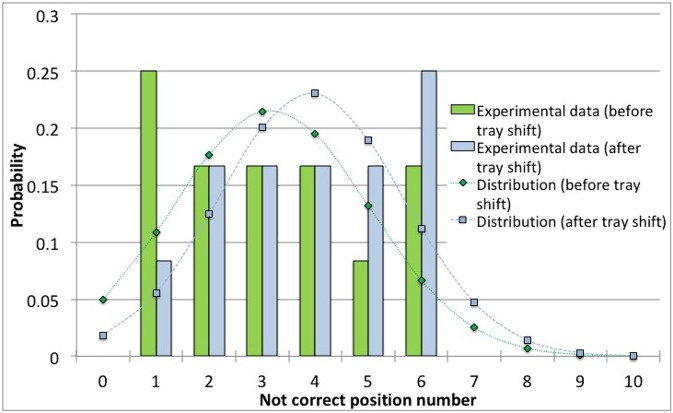
Observed data and normal probability distribution representing the incorrect positioning of thermocouples in the 4R (16 mm diameter) vials, before and after the shift of the tray.

**Figure 6:**
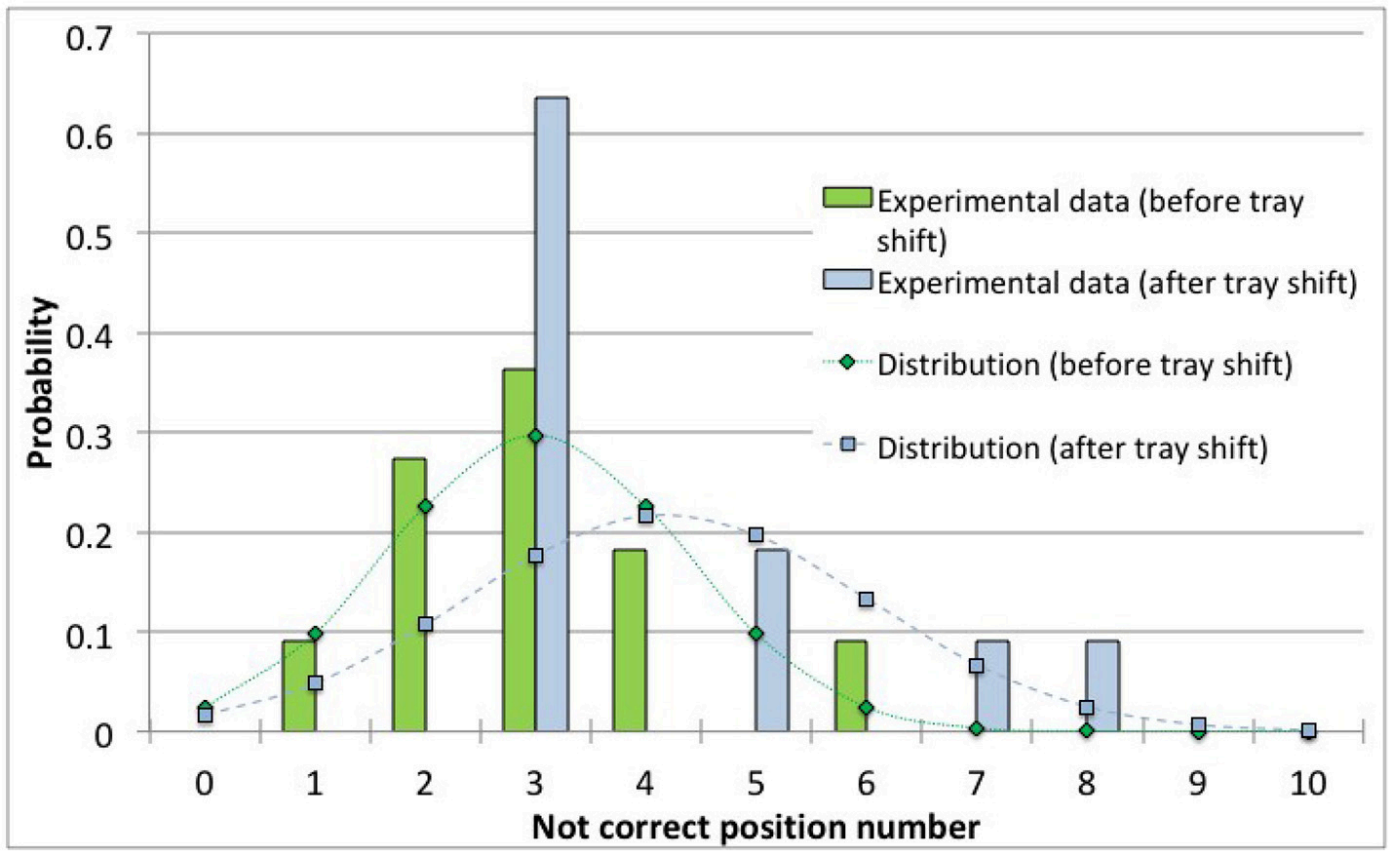
Observed data and normal probability distribution representing the incorrect positioning of thermocouples for the 10R (24 mm diameter) vials, before and after the shift of the tray.

Figure 5 show data and distribution for 4R vials. The histograms represent the observed number of incorrect positions both before and after tray shifts, that was approximated with a normal probability distribution, as discussed above. Similarly, Figure 6 show the results obtained for 10R vials. The parameters of the normal probability distributions are summarized in Table 5: the mean value and the standard deviation have been used to characterize the probability for risk estimation purposes, as discussed in the following sections.

**Table 5:** Parameters of the normal probability distribution approximating the observed numbers of thermocouple positioning errors for 4R and 10R vials.

**Vial type**	**4R**		**10R**	
	**Before shift**	**After shift**	**Before shift**	**After shift**
μ	3.17	3.92	3.00	4.18
σ	1.85	1.73	1.34	1.83

### Effects on the freeze-drying process

**Figure 7:**
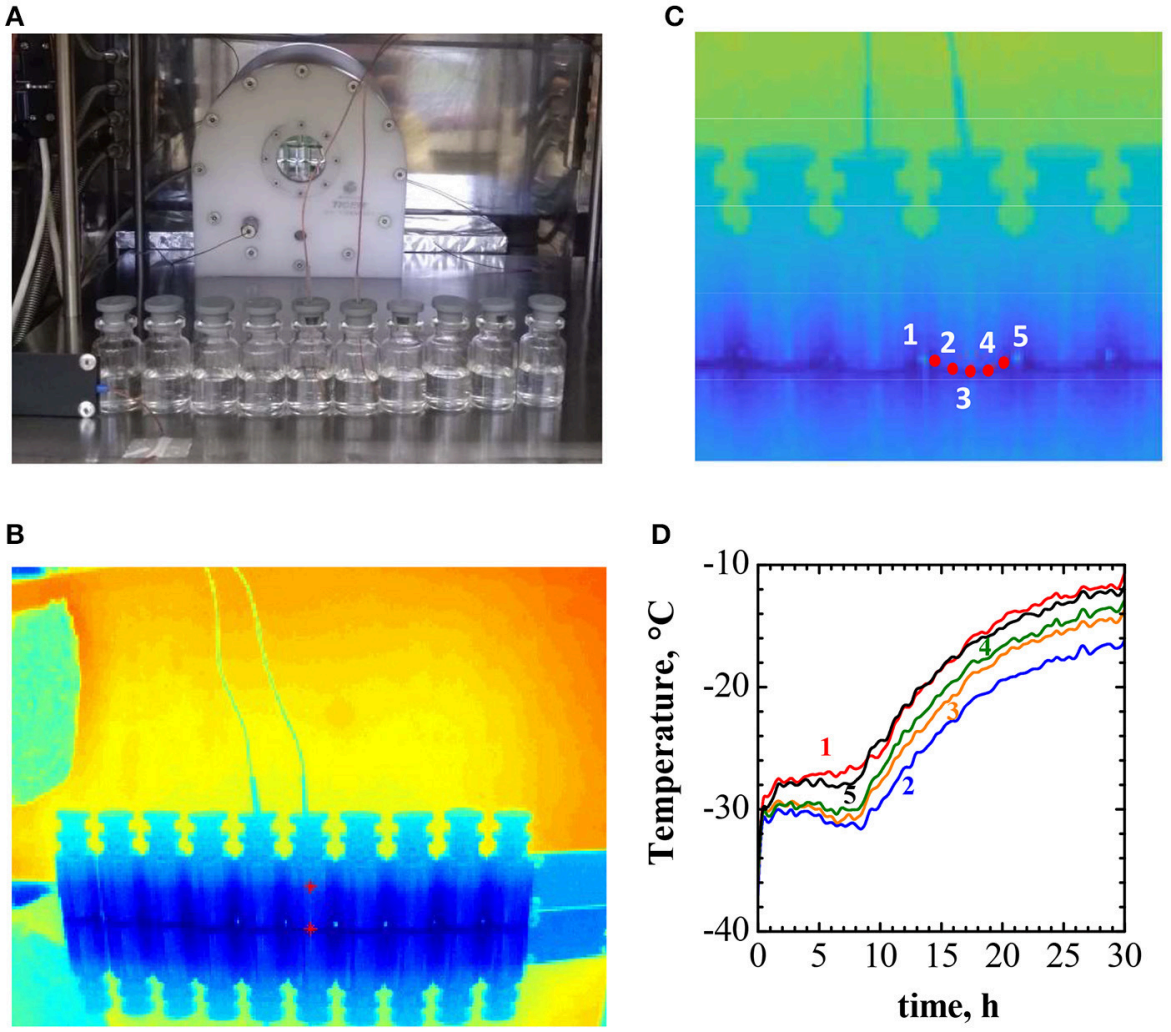
Example of experimental evaluation of temperature gradients in vials during the drying process. **(A)** The experimental set up, showing the row of 10R vials containing 10% w/w sucrose solution, with a thin-wire thermocouple inserted in two of them, and the IR thermal camera in the background. **(B)** The IR camera image. **(C)** Detail of the previous image, showing the five points where the temperature is evaluated. **(D)** Time evolution of the temperature in the five positions of the vial equipped with a thermocouple shown in **(C)**.

To exemplify the effect of the incorrect positioning of the thermocouples in the vials on the freeze-drying process of a pharmecautical product, the freeze drying of a 10% solution of sucrose in water has been taken as a text case. 10R vials containing 5 ml of solution, processed at 20 Pa and with a −20°C shelf temperature have been considered. To evaluate the temperature gradients across the vial, and thus estimate the maximum error in the thermocouple reading caused by uncorrect positioning, the set up shown in Figure 7A (using a row of ten 10R vials) and the procedure described by the compound representation given in Figure 7 has been adopted.

Figure 7B shows the thermographic image of the setup, obtained by the IR thermal camera, mounted in the freeze-drying chamber (as shown in Figure 7A). The detail of the IR image, in Figure 7C, shows the positions where temperature is recorded; the temperature evolution profiles are shown in Figure 7D. Figure 7D evidences that between the bottom-center of the vial, where the thermocouple is supposed to be, and the vial wall, where quite often the thermocouple is placed, the temperature difference may be higher than 1°C. In the case considered, as a consequence of chamber walls radiation, convective heating and conduction in the vial wall, higher temperature are observed peripherically.

As stated above, the collected information can be used to support the risk-based decision making. In fact, as proposed in Bosca et al. (2017), it is possible to define a tolerability profile for the risk of exceeding the limit temperature. The risk can be defined based on the basic equation for technological risk *R* = *p* × *M*, where *p* is the probability of occurrence of the unwanted events and *M*, the magnitude, as in Equation (4) for the freeze-drying case study:

(4) $$\begin{array}{r} R=\int_{T_{limit}}^{\infty }p\left(T_{i}-T_{limit}\right)dT_{i} \end{array}$$

where *p* is the probability that the temperature is higher than the limit temperature, *T*_*i*_ is the measured temperature, *T*_limit_ is the limit temperature. The consequences in the risk equation are thus intended in terms of overtemperature with respect to limit temperature.

Let's consider, as an example, the tolerable risk profile in Table 6, that has been hypothetised for the specific case, according to the approach detailed in Bosca et al. (2017); of course, this should be adapted in case of different applications on the basis of the experience of process supervisors. According to Equation (3) this would bring to a value of tolerable risk of 0.11°C. Taking into account the probabilities obtained from the operator data observed and reported in the section Operational Error Estimation for 10R vials, a Δ*T* between the center and the side of the vial of 1°C, and a limit temperature for sucrose freeze-drying of *T*_*max*_ = −32°C, the following consideration can be drawn. If the temperature measured from the thermocouple is −33°C, there is a probability of 0.53 that this is the measure in the correct central position, corresponding to a lateral temperature of −32°C, and of 0.47 that the temperature refers to the lateral position. With this temperature reading, in no case there will be a risk of exceeding the limit temperature, independently on the thermocouple positioning.

**Table 6:** Tolerable risk profile.

***T_*i*_* – *T_*max*_*, °C**	***p***
1	0.1
2	0.01
3	0.001
4	0.0001

If the temperature measured from the thermocouple is −32°C, there is a probability of 0.53 of having a lateral temperature of −31°C, and of 0.47 that the temperature corresponds to the lateral position, and thus is the maximum value in the product. Thus, the risk of exceeding the limit temperature will be a not acceptable value of 0.53°C.

**Figure 8:**
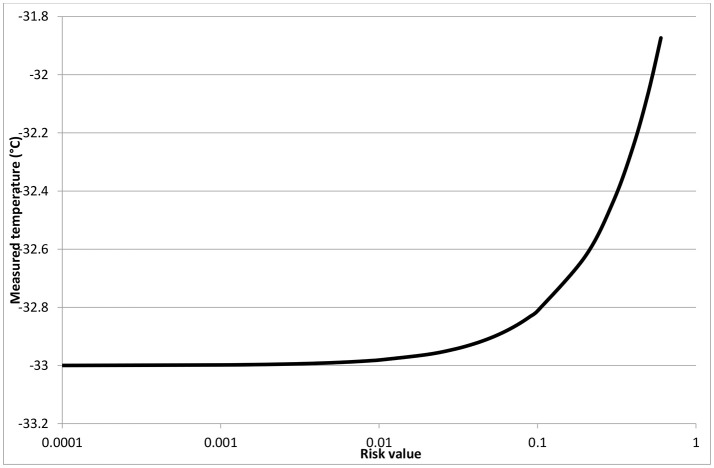
Risk profile for the sucrose freeze-drying in the case under study.

Being able to assess the level of risk of exceeding the maximum allowed temperature, even in case of operational errors in positioning the thermocouples, as summarized in Figure 8, allows determining the safety margin to be adopted in setting process temperatures; it must be evidenced that the safety margin strongly impacts on the duration of the freeze-drying cycle and thus on its cost, and thus its value must be optimized considering both risks and cost, as discussed in Fissore et al. (2012). In the case of the sucrose freeze-drying here considered, where the correct positioning would correspond to the minimum measured product temperature, the maximum temperature that the thermocouples could read, assuring a tolerable risk of exceeding the limit temprature, is −0.33°C. This value is sligthly higher that the one that would be estimated neglecting the possible positioning error, thus allowing more efficient operating conditions. More critical would be the case where the measured temperature, as a consequence of positional errors, is lower than the actual one; this can occur in case of different heating conditions, or if the thermocouple tip is lifted vertically.

## Conclusions

The above described experimental investigation has demonstrated that the operational errors in thermocouple positioning are a cause of uncertainty in the temperature measurement and, thus, beside the uncertainty of the temperature readings, also this effect has to be accounted for when using thermocouples for process monitoring (and, also, in a control loop). Both commission and omission error mechanisms have to be taken into account, and this could bring to a probability of erroneus positions within 30 and 50%. Therefore, a probabilistic distribution of product temperature may be associated to each temperature reading, thus resulting in a risk value that the temperature limit of the product being freeze-dried is trespassed.

This is a first contribution to the evaluation of the uncertainty in the temperature measurements, that will strongly affect both model parameters, and also drying time, estimated from temperature measurement, and to the assessment of their reliability, in the mainframe of a risk based approach to the freeze-drying process control. Further work will focus on the uncertainty of the model parameters and estimation of drying time, and on the evaluation of other measurement errors, including those determined by the presence of the sensing element itself, and its interaction with the sample. In fact, it must be reminded that the presence of the sensor may affect the degree of supercooling in the freezing stage and, thus, the size of the ice crystals, although this effect is not highly relevant in non-GMP (Good Manufacturing Practice) conditions, e.g., at lab-scale.

Thermocouples inserted in the product have been considered in this work, as they are the cheapest, simplest to use, and more widely used devices in laboratory practice. The possibility of placing the thermocouples outside the vial, e.g., through plasma sputtering, that allows embedding thin film sub-micrometric temperature probes in the glass wall, was also proposed and validated experimentally (Grassini et al., 2013). By this way it is also possible to realize an array of thermocouples for a more accurate process monitoring (Parvis et al., 2014), even if a model will be necessary to estimated the product temperature, and other sources of uncertainty will arise (for example, shrinkage of the product, that modifies the wall-product conductivity. The comparison of accuracy, precison and reliability of the different technical solutions, considering also possible effect of partial collapse and shrinkage, will be caried out in a future work, weighing strength and weakness of different technical solutions.

Different types of device and measuring technologies have been described in the Introduction, with their adavantages and limitations; a similar analysis can be applied also to them, to identify sources of measuring error and quantify risk; anyway, among those that allow measuring of point temperatures in single vials, the miniaturized thermocuples seem to be the less invasive and those that allow a more accurate measurement, and for this reason are generally preferred in laboratory work.

Very promising are the techniques based on multivariate image analysis for temperature monitoring, using thermal cameras as in the example shown here for estimating the thermal gradients, and work is ongoing to validate the procedure and improve reliability (Colucci et al., 2018).

## Author contributions

AB and MD raised the problem to be addressed: the impact of human factors on freeze-drying process control. GB and MD devised and planned the tests to be carried out for human error estimation, whose results have been elaborated on and interpreted by GB. AB, MD, and GB developed the discussion about the risk to be considered and the potential impact of human errors on the freeze-drying process. All of the authors participated in the paper writing and editing.

### Conflict of interest statement

The authors declare that the research was conducted in the absence of any commercial or financial relationships that could be construed as a potential conflict of interest. The handling Editor declared a shared affiliation, though no other collaboration, with the authors.
